# Forward head posture associated with reduced cardiorespiratory fitness in psychotic disorders compared to autism spectrum disorder and healthy controls

**DOI:** 10.1038/s41598-024-67604-7

**Published:** 2024-07-26

**Authors:** Ilona van de Meent, Lisanne Koomen, Renske de Boer, Lonneke Le Clercq, Dieuwertje Janssen, Mikel Boute, Arija Maat, Edwin van Dellen, Wiepke Cahn

**Affiliations:** 1 Department of Psychiatry, Brain Center at the University Medical Center Utrecht, Heidelberglaan 100, 3584 CX Utrecht, The Netherlands; 2 Graduate School of Life Sciences, Utrecht University, Utrecht, The Netherlands; 3 Lister, Utrecht, The Netherlands; 4 Human Movement Science, Vrije Universiteit Amsterdam, Amsterdam, The Netherlands; 5 Department of Psychiatry, Spaarne Gasthuis, Haarlem, The Netherlands; 6 Altrecht, Utrecht, The Netherlands

**Keywords:** Psychotic disorders, Autism spectrum disorder, Forward head posture, Craniovertebral angle, Cardiorespiratory fitness, Åstrand bike test, Psychiatric disorders, Skeleton

## Abstract

Individuals with psychotic disorders often lead sedentary lives, heightening the risk of developing forward head posture. Forward head posture affects upper cervical vertebrae, raising the likelihood of daily discomforts like skeletal misalignment, neck pain, and reduced cardiorespiratory fitness. Improving cardiorespiratory fitness in psychotic disorders is relevant, given its proven benefits in enhancing physical and mental health. This study investigates forward head posture by measuring craniovertebral angles in psychotic disorders and the relationship with reduced cardiorespiratory fitness. To determine whether forward head posture is specific to psychotic disorders, we also included individuals with autism spectrum disorder and healthy controls. Among 85 participants (32 psychotic disorders, 26 autism spectrum disorder, 27 healthy controls), photogrammetric quantification revealed a significantly lower mean craniocervical angle in psychotic disorders compared to autism spectrum disorder (*p* =  < 0.02) and the healthy control group (*p* =  < 0.01). Reduced craniovertebral angle is related to diminished cardiorespiratory fitness in psychosis (R^2^ = 0.45, *p* =  < 0.01) but not in other control groups. This study found reduced craniovertebral angles, indicating forward head posture in psychotic disorders. Moreover, this relates to diminished cardiorespiratory fitness. Further research is needed to examine the underlying causes and to investigate whether this can be reversed through physical therapy.

## Introduction

Individuals with psychotic disorders (PD) are more likely to lead sedentary lifestyles, which include physical inactivity and spending 8–13 h per day sitting^1–5^. Physical inactivity increases the risk of developing a forward head posture (FHP), a severe condition affecting the upper cervical vertebrae, characterized by sagittal protrusion of the head^6,7^. It is typically identified by a craniovertebral angle (CVA) measuring less than 48–50 degrees^8^. Disturbances in this region can lead to negative daily impacts on the body, such as causing skeletal misalignment, difficulty in breathing and swallowing, as well as leading to pain and headaches^9–11^.

Studies of the upper-body region in PD showed disparities in the neck and head, described as a “hanging of the head”. In a motion capture study on gait and posture, Martin et al. observed this phenomenon in 18 out of 20 schizophrenia patients^12^. Additionally, Cristiano et al. found sagittal head tilt in patients with schizophrenia^13^. In our previous research on gait analysis, we showed that, compared to controls, PD patients more often use upper-body initiation when starting locomotion instead of leg initiation, specifically with the head, as well as increased sagittal bending of the upper body and head during locomotion^14^. However it remains unknown whether an FHP is specifically observable in PD compared to other psychiatric diagnoses, such as autism spectrum disorder (ASD), where sedentary behavior is similarly prevalent among the population^15,16^.

Furthermore, FHP can adversely affect cardio-respiratory fitness (CRF) by weakening the trapezius muscles and obstructing normal inspiration^6,11,17^. Maden et al. measured CRF with the 6-min walking test and found that an FHP with an CVA less than 48° adversely impacts pulmonary capacity within their sample group of healthy participants^18^. Morphological changes due to FHP contribute to reduced CRF and exercise inefficiency^19^. Moreover, FHP affects vital capacity and diaphragm movement, potentially leading to challenges in achieving normal lung expansion. Consequently, this may reduce respiratory function, total lung capacity, and gas partial pressure^20^.

CRF is significantly diminished in PD. Previous studies have demonstrated that improvements in CRF can mitigate alterations in brain volume in individuals with schizophrenia, while also enhancing overall physical health, social functioning, quality of life, and reducing psychotic symptoms^21,22^. Evaluating FHP in relation to CRF in this population may therefore be relevant^4,5,23,24^.

Moreover, the possible causes of an FHP have not been explored in individuals with PD. It is hypothesized that, in addition to physical inactivity, other clinical factors may increase the likelihood of postural alterations in PD. For instance, the use of antipsychotic medication, often associated with extrapyramidal movement disorders, as well as the severity of psychological and psychiatric symptoms, could play a role in developing an FHP^25–27^.

In this study, we aim to investigate FHP in PD by calculating the CVA. Additionally, we will examine if FHP is associated with diminished CRF in PD, measured with the Åstrand Bike Test for submaximal VO_2_. To determine whether this phenomenon is specific to PD, the measurements will not only be assessed in healthy controls, but also in individuals with ASD receiving the same supported-care as patients with PD. Additionally, if the CVA proves to be significantly reduced in PD, further investigation into the relationship with antipsychotic usage, the most prevalent motor disturbance(s), physical activity and number of psychiatric symptoms will be conducted through post hoc analyses using linear regression.

## Method

### Study design

A comparative cross-sectional study was conducted as part of the Psychiatric Human Movement Analysis study, an add-on study of the MUVA project (Moving together for social reintegration in people with severe mental illness) from the University Medical Centre Utrecht (UMCU) in collaboration with Lister and Altrecht, organizations who both provide care for people with a psychiatric vulnerability in the Utrecht region of The Netherlands^28^. Participants visited the UMCU for physical measurements and questionnaires. The MUVA project was registered prospectively in The Netherlands Trial Register (NTR) as NTR NL9163 on December 20, 2020, https://trialsearch.who.int/Trial2.aspx?TrialID=NL9163. This study was conducted according to the principles outlined in the Declaration of Helsinki (amended version in October 2013) and followed the guidelines of Good Clinical Practice from the European Medicines Agency (ICH E6, R2) as well as the regulations of the Medical Research Involving Human Subjects Act (WMO).

### Study population

The groups were matched according to age, sex, and BMI to minimize potential confounding variables and improve comparability between the groups. Participants with PD and ASD received the same professional-supported care and were recruited from Lister and Altrecht^28^. A supported housing organization aids individuals with severe mental illness in their rehabilitation by providing assistance across various aspects of their lives, including housing, daily activities, (voluntary) work, social contacts and social inclusion, and financial management. Within this setting, psychiatric treatment is not provided; instead, it is administered by general practitioners or psychiatrists from other organizations. Patients are assigned a key mental health worker who is responsible for the guidance plan and rehabilitation support^28^.

According to the study protocol of MUVA, all patients with severe mental illness were invited by their key mental health worker who provided information about the study^28^ . Participants received information through a letter, which was attached to the consent letter, and had the opportunity to contact the research team. All individuals in the MUVA cohort diagnosed with PD were included in this analysis^28,29^. To determine if FHP is specific to PD among psychiatric diagnoses, individuals with ASD were similarly selected in a second control group. This selection was based on comparable clinical characteristics such as sedentary behavior, lower quality of life, and significant psychological and psychiatric symptoms^16,30^. Moreover, prescribing antipsychotic medication also occurs in the ASD population, including for managing symptoms like sensory sensitivity, irritability, and repetitive behaviors^31^ . Furthermore, ASD constitutes the second-largest group within the sample, meeting the required statistical power as determined by the sample size calculation (for more information on the calculation, refer to Sect. “sample size estimation” in the “Methods” section).

Adult (> 18 years) participants with PD or ASD diagnosis according to the DSM-V were included. Inclusion criteria were active symptoms during the last two years before inclusion and needing professional care with the prospect of having ongoing support from Lister or Altrecht during the study period. Individuals with bipolar I disorder were included if they experienced recurring psychotic episodes. Treatment with psychotropic drugs such as antidepressants, antipsychotics, benzodiazepines, and mood stabilizers were allowed in this study. Patients without medication were also included. Participants were excluded if they were unable to give informed consent or were unable to read and speak Dutch.

Participants for the healthy control group were recruited through social media and e-mail. The research and communication department of the UMC Utrecht gave consent for using social media and e-mail to recruit participants. Recruitment also took place by distributing flyers outside the participating research organization. Male and female participants were included if they did not have a psychiatric diagnosis, if none of their first-degree relatives had PD or ASD, and if they were ≥ 18 years old. Participants were excluded if they used psychotropic medication, if they were unable to give informed consent, or if they were unable to read and speak Dutch.

### Experimental protocol

Participants were invited to the UMCU for the physical measurements. The questionnaires were administered through interviews with members of the research team.

Physical and social demographic descriptive characteristics: Body length and weight were measured to calculate the body mass index (BMI). Trained research assistants conducted the Sint Hans Rating Scale to outline the occurrence and severity of potential extrapyramidal motor disturbances, including akathisia, dyskinesia, dystonia, and parkinsonism. Psychotropic medication use was assessed through records provided by the treating psychiatrist or general practitioner of the participant. In instances where participants did not grant permission for access, they were requested to bring a most recent printed copy of their medication prescription list. The Sint Hans Rating Scale was administered to assess potential extrapyramidal movement disorders that are associated with the use of psychotropic medication^32^.

**International Physical Activity Questionnaire—short form (IPAQ-SF)** is a self-reported questionnaire that measures physical activity levels expressed in Metabolic Equivalent of Task (METs) in minutes spent on physical activities, including walking, moderate-intensity activities, and vigorous-intensity activities, over 7 days before the participant visits the UMCU for the assessments^33^ .

**The Brief Symptom Inventory (BSI)—Dutch validated version** is a self-reported questionnaire designed to measure psychiatric symptoms to assess a broad range of psychological symptoms and can be applied for screening and assessing mental health in various populations. Respondents rate the extent to which they have been suffered from each symptom on a Likert scale (e.g., from 0 to 4). The nine domains concern; Somatization, Obsessive–Compulsive, Interpersonal Sensitivity, Depression, Anxiety, Hostility, Phobic Anxiety, Paranoid Ideation and Psychoticism^34^.

**The World Health Organization Quality of Life (WHO_QOL) questionnaire** is a broad-ranging assessment that aims to capture subjective well-being and life satisfaction. The four domains concern; Physical Health, psychological health, social health, and environment^35^.

**Photometric measurements of the craniovertebral angle regarding forward head posture** The participant was invited to stand on a fixed mark placed on the ground and face the research assistant. Photos of the participant’s right side of the participant were taken in sagittal view, using a Sony camera HDR-CX240 which was also placed on a fixed point. Participants wore their hair in a ponytail or bun to prevent their hair from covering their neck. Short hair had to be put behind the ears. A snapshot of the obtained video footage was used to analyse the natural head posture of the participant^36,37^ The natural head posture is defined as “a standardized and reproducible position, of the head in an upright posture, the eyes focused on a point in the distance at eye level, which implies that the visual axis is horizontal”^37,38^*.* A snapshot could be used if; the participant did not move, the participant stood straight up, the participant looked straight ahead while standing 90 degrees turned in respect to the camera, the video frame had good quality, the ear of the participant was visible and the contour of the neck and shoulders of the participant were visible^37,38^*.*

**Figure 1:**
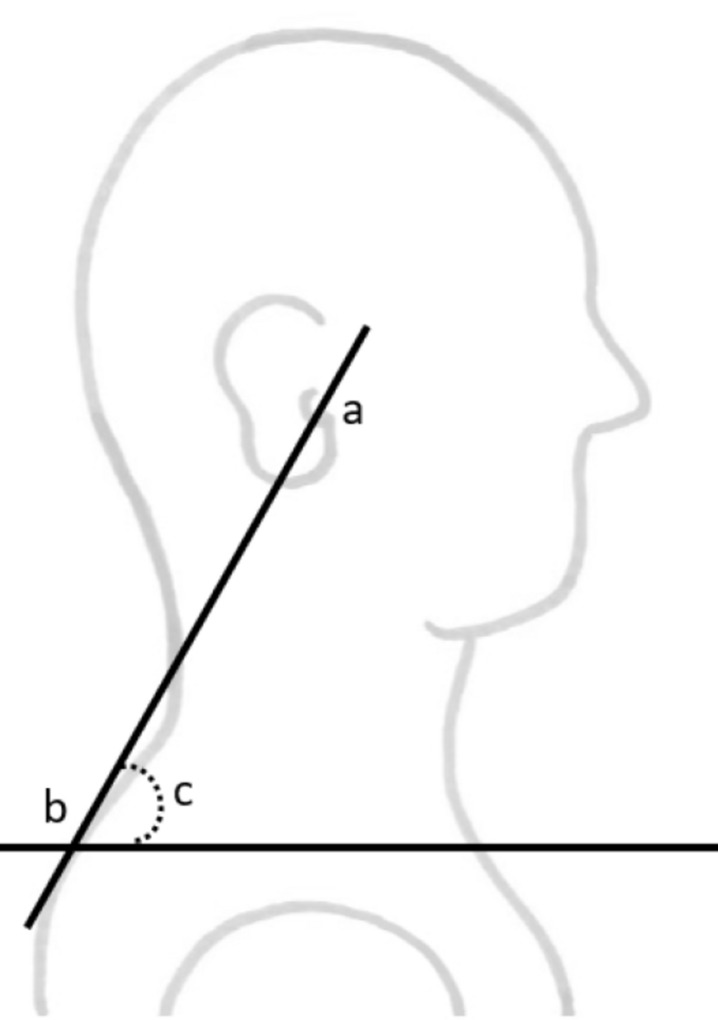
The craniovertebral angle, ^a^tragus of the ear, ^b^spinous process of C7, ^c^craniovertebral angle.

The head position was assessed afterward by measuring the CVA and was considered indicative FHP when the angle was less than 48–50 degrees^39^. The primary reason for selecting this method was its non-invasive nature, as it eliminates the need to place the head in a framework or apply markers to the skin. The CVA is defined as the angle between two lines. The first line extends from the tragus of the ear to the C7 vertebra of the spine, and the second line is a horizontal line passing through the C7 vertebra (Fig. 1). A line was inserted in the snapshot, extending from the tragus of the ear to C7 of the spine, and the height and width of the line were measured in PowerPoint. The angle was then calculated using the inverse tangent of the line inserted in the snapshot^6,7^.

**Cardiorespiratory fitness V̇O**_**2**_** (Submaximal), measured with the Åstrand Bike Test** measuring oxygen consumption during intense exercise, is expressed as milliliters of oxygen per kilogram of body weight per minute (ml/kg/min) for the CRF^40,41^. The V̇O_2_ submaximal version is selected because individuals with PD and ASD are recognized to experience limitations in performance due to factors such as pain or fatigue rather than exertion. Therefore, maximal exercise testing is often contraindicated.^42,43^. To determine the target heart rate for the Åstrand Bike Test, the participant's resting heart rate was calculated using the Karvonen Formula^44^. The assessment adhered to the official safety protocol, involving a medical anamnesis administered by research assistants and supervised by a medical professional. The participant's well-being during the test was monitored using the OMNI scale, especially suitable for individuals with ASD and PD due to its visual support^45,46^. The Åstrand Bike Test involved cycling on a cycle ergometer (Brand: Tm Tech Med) for approximately six to eight minutes with a consistent peddling rate of approximately 45 rounds per minute, adjusting resistance based on heart rate and well-being. The Polar H9 heart rate sensor with the Polar Beat application tracked the participant's heart rate. The ambient temperature during all measurements was maintained at 19 degrees Celsius. V̇O_2_ submaximal was calculated using the Åstrand & Rhyming Nomogram. Exclusion criteria for initiating or continuing the bike test and the subsequent analysis included if; the medical history revealed a contraindication, the participant declined to commence or wished to discontinue during the test, an omni-score 9 or more was reported, an excessively high heart rate occurred, or the target heart rate was not achieved, if the paddling rate per minute was too slow or too quick, or if the participant felt or appeared unwell or uncomfortable. When calculating total scores, age, BMI, and sex were considered.

### Data treatment and statistics

All statistical analyses were conducted using IBM SPSS version 26.0.0.1. The data underwent Shapiro–Wilk tests to verify normality.

#### Sample size estimation

To estimate the necessary sample size for primary outcome analysis of a potential FHP, a one-way Analysis of variance (ANOVA) was conducted to achieve 80% statistical power, significance level (α) of 0.017 (0.05/3 group comparisons), and a standard deviation of 10 degrees based on publications using similar methods^13,37^. This resulted in a minimum sample size of n = 75, with a minimum of 25 participants in each group.

#### Descriptive data

The control groups were independently assessed for statistically significant differences compared to the PD group. One-way ANOVAs were used to assess for differences. If data did not follow a Gaussian distribution, Kruskal–Wallis tests were conducted, and for categorical data. Fisher’s exact test was employed if the cell count was fewer than 6.

#### Calculation of psychotropic medication

The defined daily dose (DDD) methodology was employed for calculating medication dose equivalence, as endorsed by the World Health Organization Collaborating Centre for Drug and Statistics Methodology from 2018^47,48^. The DDD is the assumed average daily maintenance dose for a drug used for its primary indication in adults^48^. DDDs are assigned only to drugs with an anatomical therapeutic chemical code (ATC)^49^. Equivalent doses to Fluoxetine (ACT N05AH03) for antidepressants, olanzapine for antipsychotics (ACT N06AB03), diazepam (N05BA01) for benzodiazepines, and valproic acid (ACT N03A601) for mood stabilizers were calculated using the ATC/DDD index 2023 of the WHO^50^.

#### Analysis of group differences in CVA and CRF

An analysis of covariance (ANCOVA) was conducted to assess group differences in CVA scores. We included age, BMI, and sex as covariates. A Bonferroni correction was applied resulting in a significance level of 0.017. In line with prior research establishing the reliability of CVA measurements, a symmetrically distributed coefficient of 1.98 was applied to accommodate potential sources of Standard Error of Measurement (SEM)^7^. Building on previous research exploring the connection between CVA and CRF, our hypothesis posits that variations in CRF can be explained by CVA. Consequently, the linear regression analysis incorporates 'CVA' as the independent variable and 'CRF' as the dependent variable.

#### Post-hoc analyses of clinical factors

Post-hoc analyses will be conducted through linear regression with age, antipsychotic medication usage, the most prevalent extrapyramidal motor disturbance(s) measured with the SHRS, physical activity measured with the IPAQ-SF, and total number of psychiatric and psychological symptoms measured with the BSI as dependent variables and the CVA as independent variable.

### Ethical approval

**Figure 2:**
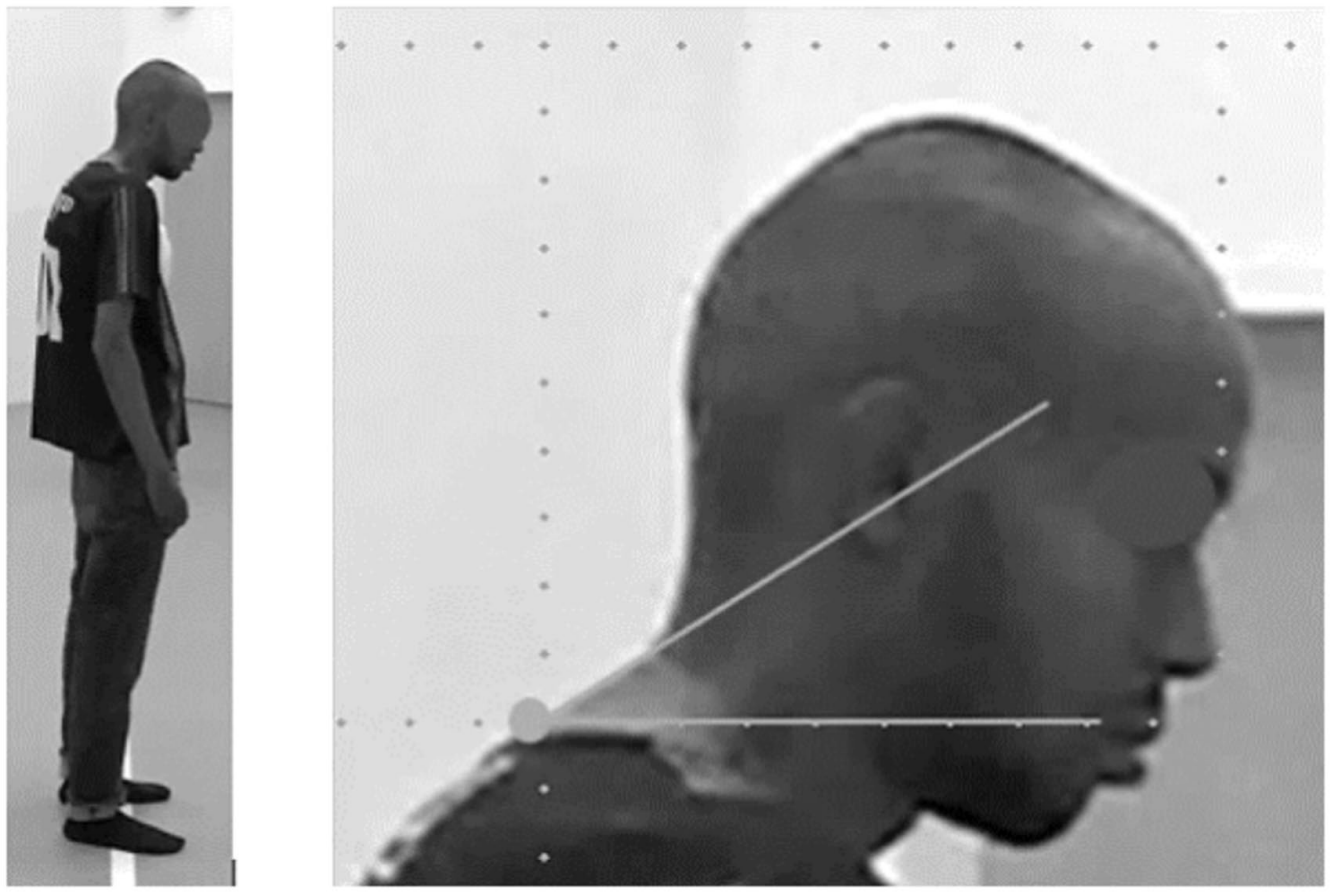
This figure displays an example measurement from the study, which shows the craniovertebral angle to be 33.05°. This figure is published with explicit informed consent regarding open-access publication. At the request of the participant, the image is slightly blurred, and the eyes appear unrecognizable.

The Medical Ethical Committee of the University Medical Centre Utrecht granted approval for this study (number: 20-628). Informed consent was obtained from all participants. Figure 2 is published with informed consent regarding the identifying image in an online open-access journal.

## Results

### Demographic results

85 participants were recruited for this study. 32 participants with PD, 26 with ASD, and 27 HC, meeting the required sample size (Table 1. provides descriptive statistics, and supplementary Table 1. offers an overview of the included PD diagnoses).

**Table 1:** Social demographic and clinical characteristics.

Descriptive statistics	n	PD n = 32	n	HC N = 27	n	ASD N = 26
**Sex,** N (%) Male	32	24 (75.00)	27	9 (33.30)**	26	20 (76.90)
**Age in years**, mean (SD)	32	46.41 (11.41)	27	47.52 (15.41)	26	37 (12.85)**
**BMI** mean, (SD)	32	27.66 (4.26)	27	25.35 (4.40)	26	27.7 (5.20)
**Educational attainment,** N (%) ^a^	30^a^		27		26	
Primary school		3 (10.00)		0 (0)**		2 (7.70)
Secondary school		11(34.38)		2 (3.70)**		3 (11.54)
VET level 1–2		5 (16.70)		1 (3.70)**		5 (19.20)
VET level 3–4		2 (6.70)		2 (7.40)**		7 (26.90)
Higher education		5 (16.70)		6 (22.20)**		2 (7.70)
University		4 (13.30)		16 (59.30)**		7 (26.90)
**Duration of illness in Years**, Median (IQR 25th–75th)^d^	15^d^	7 (4–14)	–	–	17^d^	5 (3–11)
**Sint hans rating scale**, median (IQR 25th–75th)	32^f^		27^f^		26^f^	
Akathisia	22	3.50 (5.50–6.00)	15	1.00 (1.00–3.00)*	22	5.00 (2.00–9.25)
Dyskinesia	17	4.00 (1.50–10.00)	7	2.00 (2.00–2.00)	13	4.00 (2.50–9.50)
Dystonia	11	3.00 (2.00–3.00)	-	–**	12	2.00 (2.00–4.75)
Parkinsonism	30	9.00 (6.00–24.25)	4	2.50 (1.25–3.00)**	23	12.00 (8.00 -16.00)
**Psychotropic medication**—DDD in mg, Median (IQR 25th–75th)^b,c^	32^i^				26^i^	
Antidepressants (*equals fluoxetine)*	8	20.00 (10.00–30.00)			5	40.00 (7.00–43.50)
Antipsychotics *(equals olanzapine)*	22	6.20 (6.20–15.00)			3	3.30 (0.60–3.50)*
Benzodiazepines *(equals diazepam)*	10	2.00 (1.80–8.50)			2	4.50 (4.00–)
Mood stabilizers *(equals valproic acid)*	7	900.00 (775.00–1625.00)			1	500.00 (–)
**World health organization quality of life**, mean (SD)	29^e^		27		24^e^	
Environment		15.70 (2.00)		18.50 (3.30)*		15.20 (2.10)
Physical health		13.90 (2.50)		17.70 (2.30)*		13.50 (2.80)
Psychological		13.70 (2.30)		16.00 (2.00)*		13.20 (2.40)
Social relationships		14.00 (3.40)		16.00 (4.00)		13.90 (3.40)
**Brief symptom iventory domains**, mean (SD)	28^e^		27		23^e^	
Total number of symptoms		21.00 (13.00)		7.00 (9.00)**		24.00 (12.00)
Anxiety^g^		9.60 (3.80)		7.30(2.10)**		10.40 (4.50)
Depression^g^		8.60 (3.00)		5.90 (1.90)**		10.00 (4.10)
Hostility^h^		6.90 (2.60)		5.70 (0.90)**		7.80 (2.60)
Interpersonal sensitivity^g^		6.60 (2.50)		4.50 (1.20)**		7.70 (2.80)
Obsession-compulsion^g^		8.90 (2.04)		6.90 (1.70)**		10.10 (4.10)
Paranoid ideation^g^		8.50 (2.90)		5.90 (1.80)**		9.30 (3.50)
Phobic anxiety^g^		8.40 (3.60)		5.30 (0.90)**		7.40 (3.50)
Psychoticism^g^		8.40 (3.60)		5.30 (1.20)**		7.40 (3.50)
Somatization^g^		8.40 (3.30)		7.70 (2.00)**		9.40 (3.80)
**International physical activity questionnaire short form**—total score in METS^h^ Median (IQR 25th–75th)	32	1073 (428–2771)	27	3426 (2055–4585)**	24^e^	1366 (873–3051)

Overview of descriptive statistics of individuals with PD = Psychotic Disorders, HC = Healthy Controls and ASD = Autism Spectrum Disorder. N = number of participants included SD = Standard Deviation. IQR = Interquartile Range. VET = Vocational Education and Training, * = *p*-value < 0.05 between PD and HC, ** = *p*-value < 0.01 between PD and ASD.

^a^N = 30 in the PD group because of 2 missing values.

^b^Some participants used more than one type of medication

^c^The DDD (Defined Daily Dose) in milligrams (MG) represents the estimated average daily maintenance dosage for a drug when used for its primary indication in adult patients, as defined by the WHO Collaborating Centre for Drug and Statistics Methodology in 2018. DDDs are exclusively designated for drugs that have been assigned an ATC (Anatomical Therapeutic Chemical) code.

^d^Missing values occurred because the onset of symptoms was not known by some participants.

^e^Missing values occurred because not all participants completed the questionnaires.

^f^Number of participants who exhibited motor disturbances or using psychotropic medication. There were no missing values.

^g^Severity of the symptom.

^h^METS Metabolic Equivalent of Task, the total score includes moderate, vigorous, and walking minutes per day over 10 days before the measurements at the UMCU.

^i^Participants who did use antipsychotic medication. Some participants used more than one type of medication.

The ASD group was younger than the PD group (*p* = 0.01), while no significant age difference was found between the HC and the PD group (*p* = 0.75). PD and ASD groups consisted of more males than the HC group (*p* =  < 0.01) with higher BMI scores than HC (*p* = 0.05). No significant difference was found between PD and ASD patients in duration of illness (*p* = 0.53) based on available data in a subset of 32 subjects with 15 PD participants and 17 with ASD.

Participants in the PD group used antipsychotic medication more often than those in the ASD group (*p* = 0.03). No differences were observed concerning the other psychotropic medication types, including antidepressants (*p* = 0.21), benzodiazepines (*p* = 0.38) and mood stabilizers (*p* = 0.25).

In the PD group, 96% of the participants exhibited one or more extrapyramidal motor disturbances, as evaluated by the Sint Hans Rating Scale, in contrast to the control groups (HC = 70%, ASD = 88%). No significant differences in the severity of the motor disturbances were identified between the PD and ASD groups. Motor disturbances were also observed in the HC group, but with a markedly lower incidence than in PD. Only dyskinesia demonstrated no statistically significant distinction between HC and PD (*p* = 0.13).

No significant differences were found between PD and ASD groups in The Brief Symptom Inventory on all domains; anxiety (*p* = 0.50), depression (*p *= 0.18), Hostility (*p* = 0.26), Interpersonal sensitivity (*p* = 0.14), obsession-compulsion (*p* = 0.23), paranoid ideation (*p* = 0.42), phobic anxiety (*p* = 0.34), psychoticism (*p* = 0.34) and somatization (*p* = 0.84). Both the PD and ASD groups scored significantly higher than the HC on all domains.

Similarly, PD and ASD groups had lower scores on all domains of the World Health Organization Quality of Life-Brief questionnaire than the HC group, encompassing physical health (*p* =  < 0.01), psychological well-being (HC: *p* =  < 0.01), social relationships (*p* = 0.01), and environmental factors (*p* =  < 0.01). PD and ASD did not differ in quality-of-life scores.

Results of the IPAQ-SF indicate that participants in the PD group exhibit the lowest scores with a median of 1073 MET-minutes, closely followed by the ASD group with a median of 1366 METs (*p* = 0.15), with a statistically significant difference of median of 3426 METs (*p* =  < 0.01) observed in the HC group.

### Results regarding FHP and the linear regression analysis with CRF

**Figure 3:**
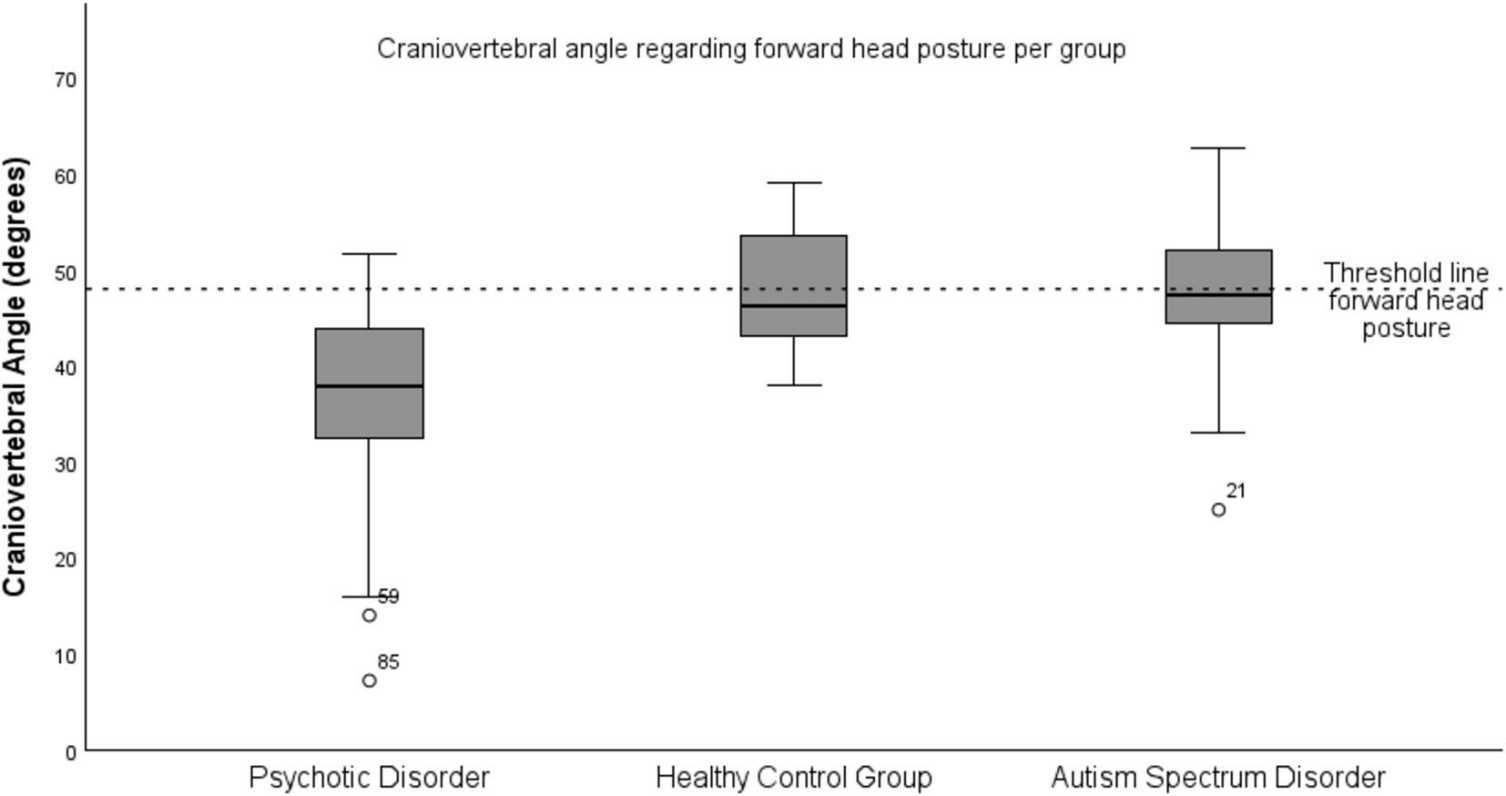
Boxplot of craniovertebral angle per group.

The CVA in the PD group was 36.07° ± 10.85° and significantly lower compared to the HC group with 48.13° ± 48.13° (*p* = 0.01) and ASD group 47.33° ± 8.81° (*p* =  < 0.02). The CVA of the ASD group did not statistically differ from the HC group (*p* = 0.88) (See Table 2, Figs. 2 and 3 for the CVA results).

**Table 2:** Physical measurements.

Physical measurements	N	Mean (SD)	F*	p*
**Craniovertebral angle** (degrees)^a^				
PD	32	36.07 (10.85)		
HC	27	48.13 (6.40)	9.33	< 0.01
ASD	26	47.33 (8.81)	4.50	0.02
**VO**_2_ **submaximal cardiorespiratory** **fitness** (ml/kg/min)^b^				
PD	15	38.70 (11.01)		
HC	21	47.50 (14.03)	3.53	0.04
ASD	15	39.51 (11.04)	1.50	0.24

PD = psychosis disorder, HC = healthy controls, ASD = autism spectrum disorder. An ANCOVA was conducted with adjustments made for ‘age’, ‘BMI’ and 'sex' in the statistical model for the results of both assessments. A bonferroni correction with a significant level of 0.017 was applied for the compairison between groups. N = number of participants included. ±  = standard deviation. * = F-ratio and p-values of PD compared to the control groups.

^a^Overview of the photometric measurements of the craniovertebral angle indicating forward head position. A symmetrically distributed coefficient of 1.98 was applied to accommodate potential sources of Standard Error of Measurement (SEM). There was no statistically significant difference between the HC and ASD groups (*p* = 0.88).

^b^The overview of the total cardiorespiratory fitness results, measured with the Åstrand Bike Test (VO_2_ submaximal). The formula used to calculate V̇O_2_ submaximal is adjusted to account for age, weight, and sex and involves milliliters per minute per kilogram of body weight (ml/kg/min).

A total of 51 participants completed the Åstrand Bike Test, distributed across PD (n = 15), HC (n = 21), and ASD (n = 15). The CRF total score of 38.70 ± 11.01 ml/kg/min in the PD group closely aligns with the average total score of 39.51 ± 11.04 ml/kg/min in the ASD group (*p* = 0.24). The HC group had a significantly higher average score of 47.50 ± 14.03 ml/kg/min (*p* = 0.04) (Table 2 and supplementary Table 2).

**Figure 4:**
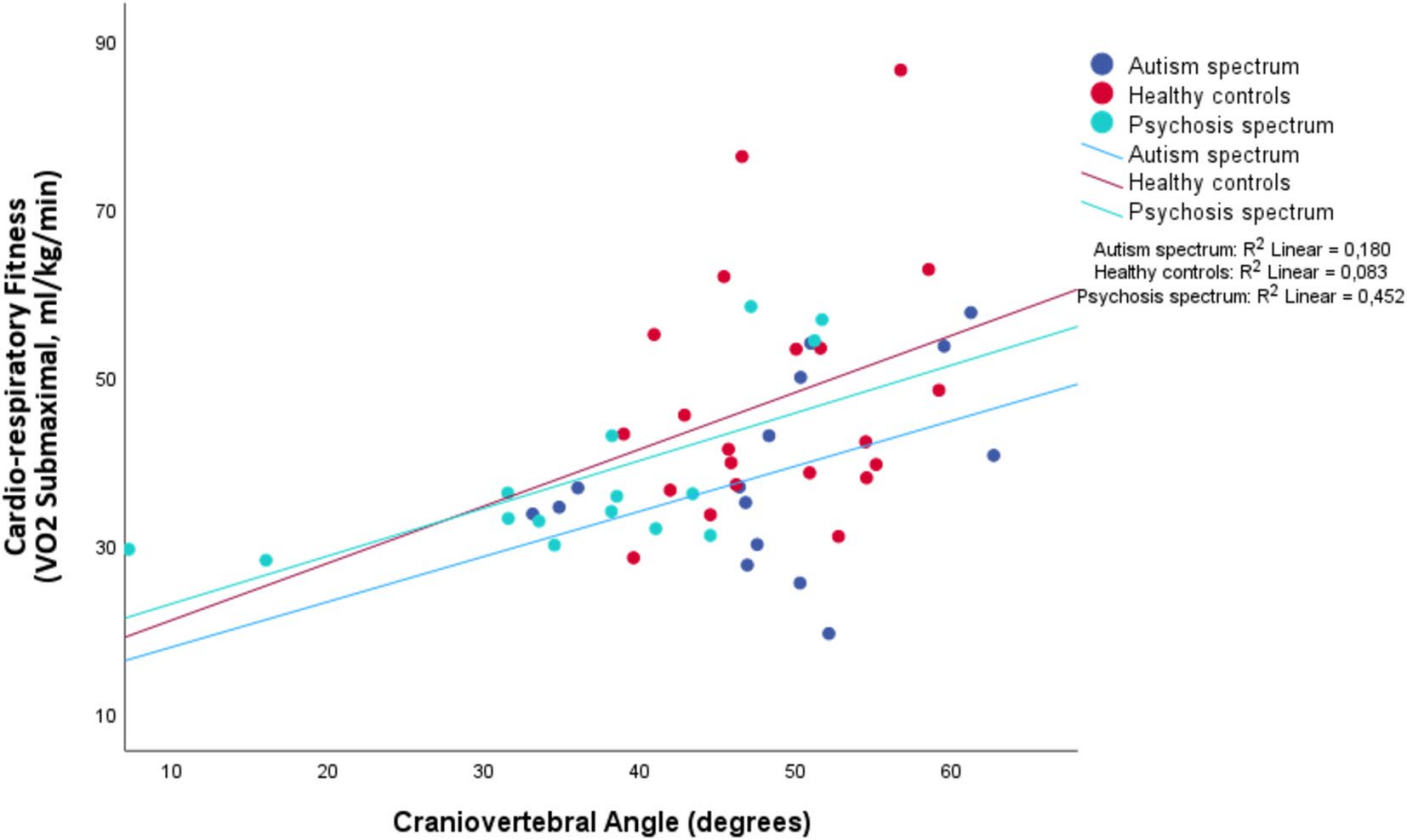
Linear regression analysis including cardiorespiratory fitness and craniovertebral angle.

The PD group exhibited the strongest correlation within the linear regression model, indicating that reduced CVA significantly accounted for variance in diminished CRF results (R^2^ = 0.45; *p* =  < 0.01); no associations were found in the HC and ASD subgroup analyses (HC: R^2^ = 0.08, *p* = 0.21, ASD R^2^ = 0.18, *p* = 0.12) (See Table 3 and Fig. 4 for the linear regression results).

**Table 3:** Linear regression analyses regarding the craniovertebral angle.

Linear regression ANALYSES—Craniovertebral angle	n	b [95% IC]	β	R^2^	*p*
1. VO_2_ submaximal cardiorespiratory fitness (ml/kg/min) ^2^					
Total	51	0.55 [0.23–0.87]	0.44	0.19	< 0.01*
PD	15	0.57 [0.19–0.94]	0.67	0.45	< 0.01**
HC	21	0.67 [ − 0.40 to 1.76]	0.29	0.08	0.21
ASD	15	0.54 [ − 0.15 to 1.23]	0.42	0.18	0.12
2. Potential explanatory factors					
Age in years PD	32	− 0.32 [ − 0.70 to 0.05]	0.67	0.09	0.09
Antipsychotic medication PD	22	− 0.02 [ − 0.38 to 0.34]	-0.03	< 0.01	0.90
Parkinsonism (SHRS) PD	30	− 0.20 [ − 0.52 to 0.13]	-0.29	0.05	0.23
Total number of psychiatric/psychological symptoms (BSI) PD	27	0.27 [ − 0.38 to 0.76]	0.48	0.02	0.49
Total score physical activity (METS -IPAQ) PD	29	37.16 [ − 17.01 to 91.34]	0.27	0.07	0.16

*1*: Overview of the linear regression analysis for the craniovertebral angle and cardiorespiratory functioning. *Linear regression is assessed with a significance level of 0.05 for the total group analysis. **given the smaller sample size in the group analysis, significance is asserted from 0.01.

*2*: Overview of potential confounding factors concerning the reduced craniovertebral angle in the psychotic disorders group. SHRS = Sint Hans Rating Scale. BSI = Brief Symptom Inventory. IPAQ = International Physical Activity Questionnaire. METS = Metabolic Equivalent of Task. significance is asserted from 0.05.

### Post-hoc analyses regarding clinical factors

Given the significant reduction in the average CVA in PD compared to the control groups, a post hoc analysis utilizing linear regression was conducted. This analysis aimed to explore clinical factors, which might elucidate the observed CVA reduction, including age (R^2^ = 0.09, *p* = 0.09), physical activity (R^2^ = 0.07, *p* = 0.17), antipsychotic medication usage (R^2^ =  < 0.01, *p* = 0.90), presence of parkinsonism (R^2^ = 0.05, *p* = 0.23), and number of psychiatric symptoms (R^2^ = 0.02, *p* = 0.49). However, none of these variables exhibited a significant relationship with the reduced CVA in the PD group (see Table 3 for the outcomes of the linear regression analyses).

## Discussion

### Main findings of this study

In this cross-sectional study, we found evidence of a significantly lower CVA in PD compared to ASD and HC (Table 2, Figs. 2 and 3). Furthermore, a relationship between decreased CVA and diminished CRF was observed in PD (Table 3 and Fig. 4). Clinical factors, including antipsychotic medication usage, parkinsonism, physical activity, and the number of psychiatric symptoms did not exhibit a significant relationship with reduced CVA (Table 3). These findings suggest that FHP may be more prevalent among individuals with PD and could be a potential contributing factor to diminished CRF in this population.

Regarding the CVA results in PD, 30 out of 32 participants demonstrated FHP, with a group mean CVA of 36.07° ± 10.85° (Table 2, Figs. 2 and 4). This is approximately 12°–14° below the 48°–50° threshold, indicating severe FHP^6,7^. This aligns with previous findings by Cristiano et al., who reported an average neck-head angle of 33.35° ± 12.5° in early-stage schizophrenia and 33.91° ± 17° in late-stage schizophrenia participants^13^.

The mean CVA results in the ASD and HC groups were both near the threshold line of FHP and significantly higher compared to the PD group (ASD: 47.45° ± 8.81°, *p* = 0.02, HC: 48.13° ± 6.40°, *p* =  < 0.01) (Fig. 4 and Table 2)^6,36^. It remains uncertain if the ASD results are representative of the ASD population due to the lack of prior studies on FHP in this group.

Furthermore, we investigated the relationship between FHP and CRF through linear regression analysis. The PD group showed CRF results in the borderline range between 'fair' and 'good,' which is better than results from similar studies where participants with PD fell into the 'poor' category (Table 2)^4,43^. However, a 50% dropout rate in the PD group, with 35.05% due to respiratory issues, may have influenced this outcome (supplementary Table 2). Nonetheless, a significant relationship between reduced CVA and diminished CRF was evident (Table 3, Fig. 4). Future research might prioritize finding ways to include individuals with respiratory issues in CRF VO_2_ max tests and assess whether adjustments strengthen the observed relationship.

The ASD group exhibited similar CRF results compared to the PD group, but no association with CVA was found. It remains unclear whether the CRF findings are representative of the adult ASD population, as, to the best of our knowledge, no research has been conducted on these topics. Given the moderately low CRF results in the ASD group, we would like to underscore the importance of further investigation. The HC group attained a classification in the ‘excellence’ category, aligning with a substantial effective oxygen uptake according to the validated protocols to measure CRF employed in this study. Also in the control group, there was no association with CVA (Tables 2 and 3, Fig. 4)^40^.

Since the mean CVA was found to be significantly lower in PD compared to the control groups, a post-hoc analysis using linear regression was conducted on clinical factors including, physical (in)activity, antipsychotic medication usage, parkinsonism, and number of psychiatric symptoms.

Regarding physical activity, the results indicate that the total number of METs from the IPAQ-SF was low, but there was no relationship with low CVA (Table 3). This finding is notable since the low CRF was related to low CVA, and low CRF levels are associated with physical inactivity^51^. A plausible reason for this discrepancy could be the small sample size of our study.

Other Clinical factors in PD that may contribute to reduced CVA, such as the use of antipsychotic medication, parkinsonism, and symptom severity, were did not significantly correlate with the observed reduction in CVA in PD (Table 3). While these factors were not explanatory in this study, they may still hold relevance to FHP. Subsequent investigations are necessary to explore these factors.

### Clinical and practical implications

Concerning the potential impact on daily life, an FHP of this nature as observed in PD can negatively affect primary body functions, such as breathing and swallowing. This arises from compressing the thorax and narrowing the trachea^17,19^. Moreover, it may cause back, neck, and head pain due to strained (upper) trapezius muscles and misalignment of the spine^10,11,52–56^.

Furthermore, individuals with PD often exhibit gait abnormalities, including disruptions in thoracic sway and balance control^12,57–59^. These characteristics are also associated with FHP and potentially align with findings in our prior study where we demonstrated that sagittal bending of the head and thorax induces disconnection between other body parts during the initiation and execution of gait^9,14,60^.

Sedentary behavior is prevalent among the PD population, and improving physical health is important for mitigating the high morbidity and mortality rates in this group^1,5,61,62^. Although physical activity did not explain the reduced CVA in PD in this study, an FHP may hinder physical activity. Sports professionals should be aware of the potential prevalence of FHP and that this condition can present extra physical challenges for individuals with PD when they begin exercising. In this context, encouraging physical activity may also be particularly relevant in maintaining a healthy posture.

A systematic review and meta-analysis showed promising results regarding corrective exercises with improvements in CVA^63^. Moreover, Morningstar investigated a novel approach (spinal manipulative therapy) in a 27-year-old male with cervical hyperlordosis, and forward head posture. Post treatment radiographs showed a reduction on FHP of 12 mm^64^. Pilates also holds potential for bone strength and flexibility as it involves muscles and skeleton exercises^65^.

FHP may also provide new insights into the potential cause for low CRF in the PD population^4,51,62^. An average CVA less than 48° adversely impacts pulmonary capacity, respiratory biomechanics, particularly the trapezius muscles, exercise inefficiency caused by the morphological changes and, vital capacity and diaphragm movement, potentially leading to challenges in achieving normal lung expansion^11,18,66,67^. Consequently, this may reduce respiratory function, total lung capacity, and gas partial pressure^20^. Although the direct relationship between forward head posture and its impact on oxygen supply to the vital organs is not fully understood, these symptoms have been identified as potential contributing factors^68^. This might be noteworthy in PD, due to the frequent occurrence of heart and vascular diseases, along with studies suggesting aberrant cerebral oxygenation^69,70^.

The necessity for interventions to correct FHP appears apparent to improve CRF in PD and although the relationship between CRF and FHP is proven, research on this topic is rare. Kim et al. found that McKenzie exercises were successful in enhancing cardiovascular fitness^71^. Dareh-deh et al. discovered within-group differences following an 8-week program that involved resistance and stretching exercises targeting diaphragm muscle activation, respiratory balance, and the number of breaths^72^.

Considering these potential clinical and practical implications, as well as the overall vulnerable physical and mental health of individuals with PD, an FHP, may extend beyond physical discomfort. This highlights the importance of identifying musculoskeletal deviations as well as a holistic approach that addresses both mental and physical well-being in PD.

### Strengths and limitations

The sample size of our study exceeded the required power for comparison. Moreover, the inclusion of individuals with ASD provided valuable insights, as they closely matched clinical variables such as symptom severity (BSI), quality of life (WHO_QOL), motor disturbances (SHRS), and physical activity (IPAQ_SF). Furthermore, we controlled for potential confounding effects by considering age, sex, and BMI as covariates. The results remained significant after these corrections.

The study has some limitations that need to be addressed. Firstly, the age range was broad, which could affect the results. Therefore, we paid extra attention to this aspect through linear regression. Secondly, there were differences in age and antipsychotic medication usage between the groups of ASD and PD. Thirdly, selection bias may have been a factor in creating the subset from the overall MUVA sample. Regarding the HC group, selection bias may have also played a role, given the higher number of females with elevated education levels and slightly lower BMI. Interestingly, dyskinesia occurred in the HC group, which is rare in the general population, and had almost similar prevalence to the PD group. However, it is not clear what caused this effect in the HC group. It is recommended to include medical history in future studies to gain a better understanding of potential risk factors and overall health.

Regarding pharmacotherapy, psychotropic medication usage, particularly antipsychotic drugs, was found to be highest in the PD group (Table 1). Although linear regression analysis did not find any significant relationship (Table 3), we would like to emphasize that it may affect motor functioning, muscle tone, and posture, which can lead to conditions such as akathisia, dyskinesia, dystonia, and parkinsonism^32,73^. Even though there were no significant differences in motor disturbances measured with the SHRS compared to the ASD group (Table 1), they could still be associated with postural differences. Therefore, we recommend conducting comprehensive studies to examine medication usage in relation to FHP in the future.

## Conclusion

This study found that individuals with PD had reduced CVA compared to the HC and ASD groups, indicating an FHP. This reduction in CVA appears to be related to reduced CRF.

On an daily basis and especially when promoting physical activities, it is advisable to consider that individuals with PD may experience physical pain, disruptions in their ability to breathe, swallow, absorb sufficient oxygen, as well as to perform movements.

Considering these potential implications in PD, further research is needed to examine the underlying causes and to investigate whether this can be reversed through physical therapy. Additionally, we would like to emphasize the importance of investigating CRF in ASD.

### Supplementary Information

Supplementary Information.

## Data Availability

The datasets examined in this study can be obtained from the corresponding author upon a reasonable request. Data are located in controlled access data storage at the University Medical Center Utrecht.
