# Supplementary material for: Forward head posture associated with reduced cardiorespiratory fitness in psychotic disorders compared to autism spectrum disorder and healthy controls
Source: Sci Rep. 2024 Jul 26;14:17143. doi: 10.1038/s41598-024-67604-7 (PMC11282316; doi:10.1038/s41598-024-67604-7)
Supplement: Supplementary file 1 — Supplementary Information. [file 41598_2024_67604_MOESM1_ESM.docx]

**Supplementary Table 1:** Overview of psychotic disorders included in this study.

| DSM-5 code | DSM 5- psychotic disorder diagnoses | Count |
| --- | --- | --- |
| 295.3 | Paranoid disorder | 2 |
| 295.7 | Schizoaffective disorder | 4 |
| 295.9 | Schizophrenia | 16 |
| 296.8 | Bipolar-2 disorder, with recurring psychotic episodes | 4 |
| 298.9 | Unspecified schizophrenia disorder | 6 |

**Supplementary Table 2:** Overview of exclusions regarding the Åstrand Bike Test

| **Reason for exclusion** | **PD**  **N (%)** | **HC**  **N (%)** | **ASD**  **N (%)** | **Total per category**  **N (%)** |
| --- | --- | --- | --- | --- |
| Unable to participate due to personal reasons. | 5 (29.40) | 2 (33.30) | 1 (9.10) | 8 (23.50) |
| Exclusion based on medical history. | 2 (11.80) | 0 | 1 (9.10) | 3 (8.80) |
| Encountered respiratory issues during the assessment. | 6 (35.30) | 2 (33.30) | 3 (27.30) | 11 (32.40) |
| Failed to attain the calculated target heart rate. | 1 (5.90) | 0 | 1 (9.10) | 2 (5.90) |
| Heart rate too high during assessment | 0 | 2 (33.30) | 3 (27.30) | 5 (14.70) |
| Declined participation in the bike test. | 1 (5.90) | 0 | 2 (18.20) | 3 (8.80) |
| Difficulty following the instructions | 2 (11.80) | 0 | 0 | 2 (5.90) |
| **Total exclusions** | 17 (50.00) | 6 (17.65) | 11 (32.35) | **34 (100.00** |
